# Long non-coding RNA COL4A2-AS1 facilitates cell proliferation and glycolysis of colorectal cancer cells via miR-20b-5p/hypoxia inducible factor 1 alpha subunit axis

**DOI:** 10.1080/21655979.2021.1969833

**Published:** 2021-09-03

**Authors:** Zijun Yu, Yeming Wang, Jianwu Deng, Dong Liu, Lingling Zhang, Hua Shao, Zilu Wang, Wenjun Zhu, Cheng Zhao, Qungang Ke

**Affiliations:** a Department of General Surgery, The Second People’s Hospital of Lianyungang, Lianyungang, China; b Department of Vascular Surgery, The Second People’s Hospital of Lianyungang, Lianyungang, China; c General Medicine, The Second People’s Hospital of Lianyungang, Lianyungang, China; d Clinical Laboratory, The Second People’s Hospital of Lianyungang, Lianyungang, China; e Department of Oncology, The Second People’s Hospital of Lianyungang, Lianyungang, China

**Keywords:** COL4A2-AS1, aerobic glycolysis, colorectal cancer, proliferation, HIF1A

## Abstract

Long non-coding RNAs (lncRNAs) have critical functions in tumorigenesis and progression of colorectal cancer (CRC). The role of lncRNA COL4A2-AS1 (COL4A2-AS1) lacks system investigation. The current study comprehensively analyzed the expression, biological functions, and mechanism of COL4A2-AS1 in CRC through performing real-time quantitative PCR (RT-qPCR), Western blot, cell transfection, cell colony assay, MTT assay, flow cytometry and dual-luciferase reporter system assays. A xenograft model of CRC was constructed to further verify the function of COL4A2-AS1 in CRC progression *in vivo*. The data revealed an upregulated expression of COL4A2-AS1 in CRC tissues and cell lines than paired adjacent tissues and normal cell line. Silencing COL4A2-AS1 inhibited proliferation, aerobic glycolysis, and promoted apoptosis of CRC cells *in vivo* and *in vitro*. However, overexpression of COL4A2-AS1 significantly promoted CRC cell proliferation and aerobic glycolysis. In CRC cells, miR-20b-5p was sponged by COL4A2-AS1 and hypoxia-inducible factor 1 alpha subunit (HIF1A). Restoration of HIF1A expression reversed the inhibitory effects of silencing COL4A2-AS1 on aerobic glycolysis and proliferation of CRC cells. The current findings showed that COL4A2-AS1 promoted the proliferation, and aerobic glycolysis of CRC cells potentially through modulating the miR-20b-5p/HIF1A axis.

## Introduction

As one of the most frequently diagnosed malignancies, colorectal cancer (CRC) also shows a high morbidity and mortality. In 2018, according to Global Cancer Statistics, the incidence of CRC accounts for 10.2% of all newly diagnosed cancer cases of the year, ranking the third highest in incidence and the second highest in mortality (9.2%) [1]. Noticeably, the incidence and mortality of CRC have remained the highest among all the cancers in the recent few years [2,3], and CRC incidence is increasing steadily in developing region, particularly in China [2]. Though the clinical treatment and basic research of CRC have been greatly improved [4,5], difficulties and challenges in diagnosis and treatment of CRC are yet to be overcome.

Multiple lncRNAs were abnormally expressed, thereby acting as vital modulators in the regulation of progression of various cancers including in CRC [6,7]. High-expressed lncRNA HOTAIR is responsible for the pathogenesis of aggressive colon cancer [8] and gastrointestinal stromal tumor [9]; NR2F2-AS1 with a high expression is predictive of a lower overall survival of patients with CRC [10]; serum B3GALT5-AS1 is low-expressed in CRC patients, which therefore could be regarded as a diagnostic biomarker for identifying CRC patients [11]. Previous research based on bioinformatics found 5 lncRNAs (AL117190.1, MEG3, COL4A2-AS1, LINC00184, and MIR22 HG) as vital prognostic factors for breast cancer patients [12]. COL4A2, alternatively known as collagen IV chain 2, shows a novel dominant G702D mutation in collagen structure domain, and mRNA level of collagen IV in CRC tissues was significantly higher than that of the corresponding tissues in healthy controls [13–15]. COL4A2-AS1 (COL4A2 Antisense RNA 1, Ensemble: ENSG00000232814) is an lncRNA. Collagen type IV alpha 2 (COL4A2) is an oncogene that facilitates the growth and metastasis of breast cancer cells [16]. Previously, it has also been found that COL4A2 activates the RhoA/ROCK pathway to facilitate the growth and metastasis of hepatocellular carcinoma (HCC) cells [17]. Also, up-regulation of COL4A2 expression shows anti-apoptotic effect on epithelial ovarian cancer cells [18]. However, currently, COL4A2-AS1 has not been studied in relation to any other cancer.

The molecular mechanisms of tumors has attracted much attention from researchers [19,20]. Tumor development is closely related to long non-coding RNAs (lncRNAs), messenger RNAs (mRNAs), and microRNAs (miRNAs) [21,22]. Tumor-related diagnostic and prognostic markers and therapeutic targets have been discovered through studying the molecular mechanisms of tumor development [22,23]. Many molecular markers for CRC have been discovered [24,25]. Wang et al. reported that lncRNA SATB2-AS1 inhibits the invasion and metastasis of CRC by suppressing the Snail transcription and epithelial-mesenchymal transformation (EMT) through SATB2 [26]; Zhuang et al. demonstrated that lncRNA MALAT1 sponges miR-106b-5p to modulate SLAIN2 and enhances microtubule mobility to facilitate the invasion and metastasis of CRC cells [27]; Shi et al. indicated that by acting as a competitive endogenous RNA of miR-144, lncRNA znfx1-as1 regulates EZH2 expression to promote tumor progression and metastasis of CRC cells [28]. LncRNA PAUPAR could positively regulate the expression of ZNF750 via repressing miR-17-5p, thus increased CRC T stage and local lymph node metastasis [29]. Noticeably, the functions and mechanisms of potential lncRNAs in CRC still remain unclear, which therefore requires further identification and characterization.

The xenograft tumor model of nude mice has been increasingly applied in tumor research. Use of nude mice allows the observation of growth of human tumor cells in living animals, and also provides chances to further study genes, proteins, RNA, and drugs. Innate lack of T cell immunity in nude mice and a lack of rejection response to xenogenous substances allows human tumor cells to find a viable carrier. This study also used nude mice to study the growth and metastasis of human tumor. It should be noted that that tumor-related research is dependent on immunity, as tumor metastasis in nude mice without T-cell immunity would be vastly different from that in humans.

In the current research, we hypothesized that LncRNA COL4A2-AS1 was an oncogenic factor in CRC, possibly acting through ceRNA. Our objective was to investigate the biological function and mechanism of COL4A2-AS1 in the progression of CRC. The current findings revealed that COL4A2-AS1 expression was greatly upregulated in the tumor tissues and cell lines of CRC. Functionally, COL4A2-AS1 could bind to and suppress miR-20b-5p expression to elevate the expression HIF1A, thereby promoting the proliferation and aerobic glycolysis of CRC cells.

## Material and methods

### Collection of CRC tissues

The tumor tissues and adjacent tissues (within 3 cm from cancer tissues) were obtained from 55 patients who were first diagnosed with CRC at The Second People's Hospital of Lianyungang . The experiments were approved by the Ethics Committee of The Second People's Hospital of Lianyungang, and the informed consent was signed by all the participants. All the samples were not treated with drugs prior to the experiments. Collection of human samples and subsequent performance of experiments were carried out following the Helsinki declaration.

### Cell culture and transfection

NCM460 cell line was obtained from a tumor-free Hispanic individual, and Human CRC cell lines (T84, SW480, HT-29 and LOVO) were purchased from ATCC. The cells were cultivated in DMEM medium containing 10% fetal bovine serum at 37°C with 5% CO_2_ in saturated humidity. HT-29 cells were identified by short tandem repeat (STR) profiling.

To overexpress COL4A2-AS1, the complete sequence of COL4A2-AS1 was cloned into pcDNA3.1 (Invitrogen, CA, USA) plasmid to produce pcDNA3.1- COL4A2-AS1. A null pcDNA3.1 vector at a final concentration of 100 nM for the transfection served as a control. ShRNAs COL4A2-AS1 (shCOL4A2-AS1#1: GAGTGGCGTCTTCATGGAATA; shCOL4A2-AS1#2: GCACTGCTCAGTGGCATTCAA), shRNA (GCAGTGGCGTCTTCATGGTTAT) and plasmid HIF1A (5ʹ-GATCTCGAGGCTTTTTCTTAATTTCATTCCT-3ʹ) were commercially acquired from Shanghai Biotend Biotechnology Co, Ltd., and used at a final concentration of 100 nM for transfection. MiR-20b-5p mimic (5ʹ-UCGUACCGUGAGUAAUAAUGCG-3ʹ) and inhibitor (5ʹ-CUACCUGCACUAUGAGCACUUUG-3ʹ) were employed to overexpress and knock down miR-20b-5p, respectively, with mimic NC (5ʹ-UUUGUACUACACAAAAGUACUG-3ʹ) and inhibitor NC (5ʹ-CAGUACUUUUGUGUAGUACAAA-3ʹ) (Shanghai Biotend Biotechnology Co, Ltd.) served as their negative controls, respectively. MiRNAs were transfected at a final concentration of 50 nM. T84 and SW480 cells (1 × 10^6^ /well) were transfected using Lipofectamine® 2000 reagent (Sangon Biotech Co., Ltd.) for 48 hours (h) at 37°C.

### Real-time quantitative PCR (RT-qPCR)

After separation of RNAs from the tissues (0.5 cm ×0.5 cm) and cells using Trizol (Invitrogen) with OD260/OD230 between 1.8 and 2.0, the RNAs were reverse-transcribed into cDNAs using TruScript Reverse Transcriptase kit (Norgen Biotek Corp.). The mRNA level of COL4A2-AS1 was tested by RT-qPCR assay using Luna® Universal One-Step RT-qPCR kit (SYBR; New England BioLabs, Inc.) in the Step OneTM Real-Time PCR System (Applied Biosystems). All-in-One™ miRNA RT-qPCR Reagent kit (GeneCopoeia, Inc.) was also used in miRNA reverse transcription and qPCR assay when needed. To determine the expression of HIF1A, SYBR Premix Dimmer Eraser kit (Takara) was employed in RT-qPCR. The expressions were calculated by the 2^−ΔΔCt^ method [30]. Primer sequences used in the experiment are listed in Table 1.

**Table 1.** Primers for qPCR

Genes	Forward (5ʹ-3ʹ)	Resvers (5ʹ-3ʹ)
COL4A2-AS1	GCAGTGTGGGATGGAGACA	ATGCCAGTGGAGTATCAGCC
miR-20b-5p HIF1A GAPDH U6	TGTCGACAAGCTTACACGA GCUAUUCACCAAAGUUGAATT TGCACCACCAACTGCTTAGC CTCGCTTCGGCAGCAC	GCTAGTCATGGTGCAAGA UUCAACUUUGGUGAAUAGCTT GGCATGGACTGTGGTCATGAG ACGCTTCACGAATTTGC

### Cell viability assay

CCK-8 kit (Beyotime, Shanghai, China) was performed to detect cell growth. Briefly, after the transfection, the T84 and SW480 cells at a density of 6 × 10^3^ cells/well were seeded into 96-well plates, added with 10 μL CCK-8 solution and incubated together at 37°C for 4 h for cell culture, which was terminated at the time points of 24, 48, and 72 h. Cell proliferation was read according to optical density (OD) values with a microplate reader (Synergy H4 Hybrid Reader, BioTek, Winooski, USA) at 450 nm.

### Colony formation

A total of 500 cells were plated into 6-well plates and incubated in a humidified incubator at 37°C with 5% CO_2_ for 14 days. Subsequently, the colonies were fixed by 10% formaldehyde for 30 minutes (min), then stained by 0.5% crystal violet (Beyotime, China) for 30 min, and finally photographed under a microscope (Olympus, Tokyo, Japan).

### Apoptosis analysis

Cell apoptosis was detected using flow cytometry with Annexin V-FITC and PI kit (Beyotime, Shanghai, China). Specifically, after cell culture for 48 h, the cells were resuspended in binding buffer, incubated with Annexin V at 37°C for 10 min, and dyed by PI. Cell distribution at different phases and apoptosis were detected by flow cytometer. Cell apoptosis was determined based on the proportion of cells stained with Annexin V-FITC positive and PI-negative or PI-positive.

### Nude mouse tumor formation experiment

Our animal assays were performed following the guide of the Institutional Animal Care and Use Committee at The Second People's Hospital of Lianyungang and 3 R (Replacement, Reduction, and Refinement) guidelines. For Xenograft experiments, consider that gender is also a biological variable that may affect the results, male BALB/c nude mice (6 weeks) were purchased (Shanghai Animal Laboratory Center, Shanghai, China), maintained in specific pathogen-free (SPF) conditions, and fed with free access to sterile mouse food and water. The mice were kept in sterile filter-top cages under 12-h light/dark cycles. For tumor construction, the mice were subcutaneously injected with 5 × 10^6^ T84 cells transfected with shCOL4A2-AS1 or shNC via flank. Tumor volumes were measured every 3 days according to the formula: tumor volume (mm^3^) = (width) × (height) [2/]. After 15 days, the mice were sacrificed and tumors were collected for the further analysis (including tumor weight calculation and HE staining, and immunohistochemistry analysis). All the mice were euthanized with isoflurane.

### Immunohistochemistry staining

The formalin-fixed, paraffin-embedded tissues were cut into 5-μm sections for immunohistochemistry staining. Then, the antigen was retrieved by citrate buffer (0.01 mL, pH 6.0), the sections were washed three times with PBS and incubated in endogenous peroxidase blockers for 10 min. The sections were incubated with antibodies overnight at 4°C and then with reaction enhancer before reacting with horseradish peroxidase-labeled anti-rabbit/mouse IgG antibody for 20 min at room temperature. Finally, the slices were prepared with DAB and analyzed under Olympus BX43 (×200).

### Western blot

Proteins were extracted through RIPA (Beyotime, China) and the concentration was determined using BCA protein detection kit (Beyotime, China). The protein samples were then separated by SDS-PAGE and electro-transferred onto PVDF membranes (Millipore, USA). The membranes were blocked with 5% skim milk for 2 h, and hatched first with specific primary antibodies overnight and then reacted with the secondary antibodies (anti-Rabbit and anti-mouse IgG). Finally, the bands were analyzed by the ImageJ software (USA).

### Prediction of target

LncRNA-miRNA interactions and miRNA–mRNA interactions were predicted by LncACTdb (http://www.bio-bigdata.net/LncACTdb/index.html) and TargetScan (http://www.targetscan.org/vert_71/), respectively.

### Dual-luciferase reporter assays

Wild-type (WT), or mutant (Mut) COL4A2-AS1 or HI1FA were transfected with miR-20b-5p into T84 cells and SW480 cells. WT or mut COL4A2-AS1 were transfected with miR-20b-5p into T84 cells and SW480 cells. Dual-Luciferase Reporter Assay System (Promega, USA) was applied to determine luciferase activities.

### RNA immunoprecipitation (RIP) assay

The cell lysates were pre-stored in RIP buffer. Magnetic beads containing positive control anti-Ago2 (ab5072, Rabbit polyclonal antibody, Cambridge, MA, USA) and the negative control anti-IgG were inserted to cell lysates using Magna RNA immunoprecipitation kit (Millipore, Billerica, USA). RT-qPCR was performed to detect the relative expressions of COL4A2-AS1 and miR-20b-5p.

### Glucose consumption and lactate production

Following the instructions, lactate production and glucose consumption were, respectively, detected by Lactate Assay Kit (Sigma–Aldrich) and the Glucose Uptake Assay Kit (Colorimetric, Abcam).

### LDH activity assay

The lactate dehydrogenase (LDH) activity was tested by LDH Assay Kit (C0017, Beyotime, China). The absorbance at the reference wavelengths of 490 nm and 600 nm was analyzed with a Bio-Rad microplate reader.

### Statistical analysis

The data were shown as the mean ± standard error of the mean (SEM). Two-group comparison was conducted using Student’s t-tests, while differences of multiple group were compared by analysis of variance (ANOVA), followed by Bonferroni’s post hoc test. The statistical analyses were carried out in GraphPad Prism 7.0. *p* < 0.05 was regarded as a statistical difference.

## Results

The current research hypothesized that LncRNA COL4A2-AS1 was an oncogenic factor in CRC possibly acting through ceRNA, and our objective was to investigate the biological function and mechanism of COL4A2-AS1 in the progression of CRC. The current findings revealed that COL4A2-AS1 expression was greatly elevated in the tumor tissues and cell lines. Functionally, COL4A2-AS1 could bind to and suppress miR-20b-5p expression to upregulate the expression HIF1A, thereby promoting the proliferation and aerobic glycolysis of CRC cells.

**COL4A2-AS1, miR-20b-5p and HIF1A expressions in** CRC

**Figure 1.**
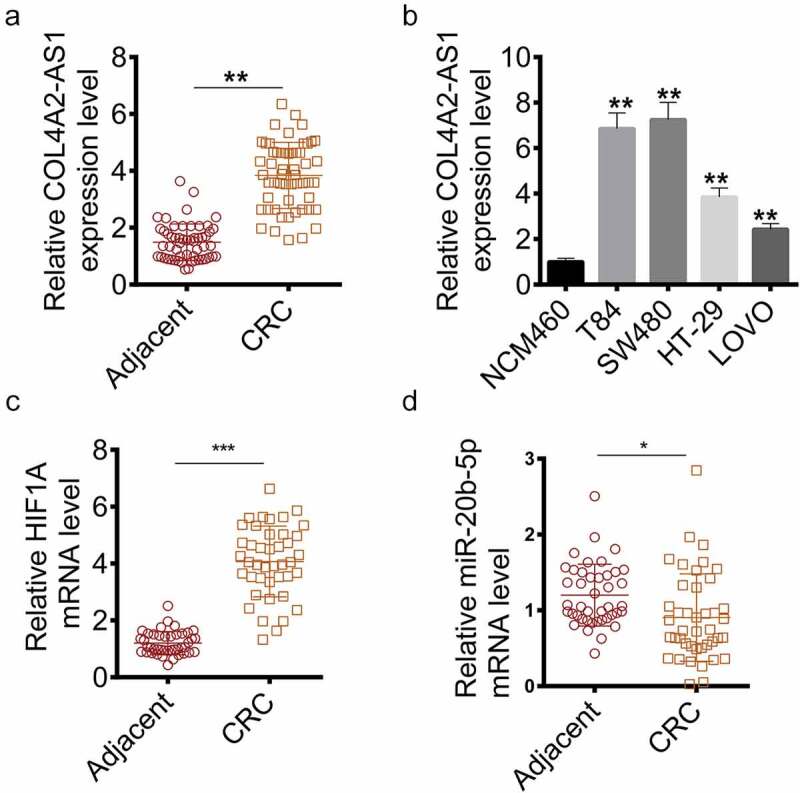
The expression of COL4A2-AS1 of the colorectal cancer tissues and cell lines were increased. (a): RT-qPCR showed that the mRNA level of COL4A2-AS1 of colorectal cancer tissues was improved. (b): RT-qPCR showed that the mRNA level of COL4A2-AS1 of colorectal cancer cell lines was improved. (c): RT-qPCR showed that the mRNA level of HIF1A of colorectal cancer tissues was improved. D: RT-qPCR showed that the mRNA level of miR-20b-5p of colorectal cancer tissues was decreased. ***P < 0.001, **P < 0.01, *P < 0.05

The COL4A2-AS1 expression in tissues and cell lines (T84, SW480, HT-29 and LOVO) was measured to determine suitable cells for studying the role of COL4A2-AS1 in CRC pathogenesis. The results showed that COL4A2-AS1 was high-expressed in CRC tissue samples compared with normal tissues (Figure 1a), moreover, similar results were also observed in cancer cell lines (Figure 1b). HIF1A was high-expressed and miR-20b-5p was low-expressed in CRC tissues (Figure 1b,c). Thus, the two cell lines (T84 and SW480) with the highest expression were used as experimental cells in the current study.

### Knocking down COL4A2-AS1 inhibited the viability and proliferation of CRC cells

**Figure 2.**
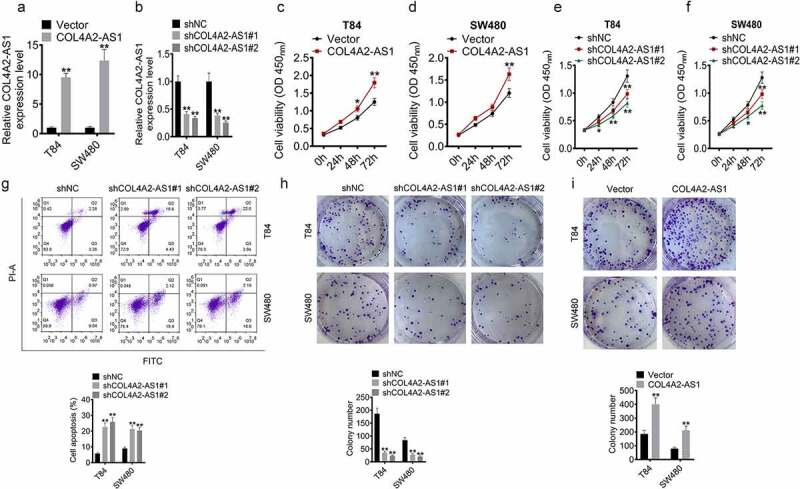
The viability and proliferation of colorectal cancer cells were inhibited by Knockdown of COL4A2-AS1. (a): COL4A2-AS1 expression was overexpressed by pcDNA3.1- COL4A2-AS1. (b): COL4A2-AS1 expression was silenced by shCOL4A2-AS1. (c–d): CCK-8 assay showed that viabilities of T84 and SW480 cells were increased by COL4A2-AS1. (e–f): CCK-8 assay showed that viabilities of T84 and SW480 cells were suppressed by shCOL4A2-AS1. (g): Apoptosis T84 and SW480 cells was improved by shCOL4A2-AS1. (h): Clone populations of T84 and SW480 cells were inhibited by shCOL4A2-AS1. (i): Clone populations of T84 and SW480 cells were increased by COL4A2-AS1. *P < 0.05, **P < 0.01

To further explore the function of COL4A2-AS1 in CRC progression, loss-of-function and gain-of-function assays were performed on T84 and SW480 cells. The data revealed that in T84 and SW480 cells, COL4A2-AS1 was obviously overexpressed by pcDNA3.1-COL4A2-AS1 but significantly silenced by sh-COL4A2-AS1 (Figure 2a,Figure 2b). Moreover, CCK8 assay demonstrated that the speed of proliferation of T84 and SW480 cells with overexpressed COL4A2-AS1 were accelerated but suppressed by sh-COL4A2-AS1 (Figure 2c–f). Annexin V-APC/PI staining showed that the proportion of apoptotic cells were clearly increased after depleting COL4A2-AS1 (Figure 2g). Also, we observed that the depletion of COL4A2-AS1 decreased clones, while more colonies formed after the up-regulation of COL4A2-AS1 expression (Figure 2h,i).

### Knockdown of COL4A2-AS1 inhibited tumorigenesis of CRC in vivo

**Figure 3.**
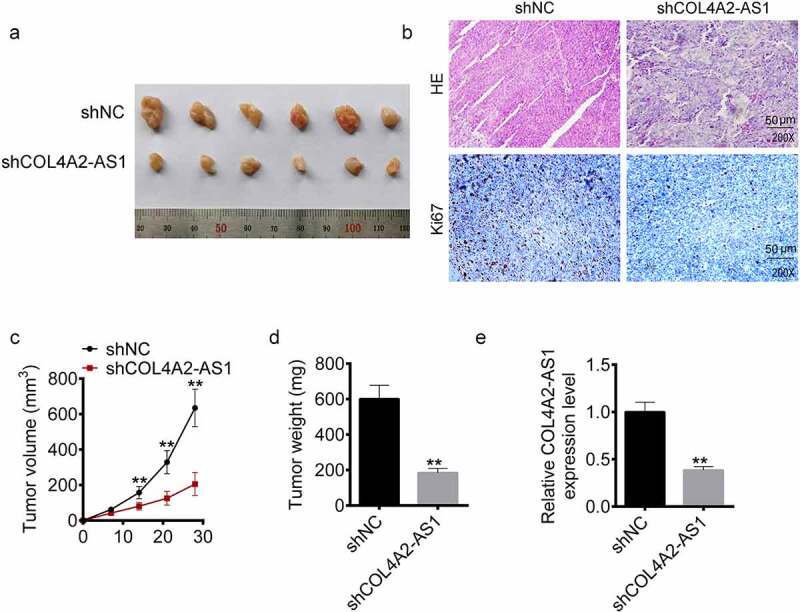
Knockdown of COL4A2-AS1 inhibited tumorigenesis of colorectal cancer *in vivo*. (a): Tumor growth was inhibited in the shCOL4A2-AS1 mouse compared with the control. (b): HE staining and Ki-67 staining. (c): Tumor size (mm^3^). (d): Tumor weight (g). €: The expressions level of COL4A2-AS1 in the tumor tissues. **P < 0.01

A xenograft model of CRC was constructed with COL4A2-AS1 knockdown (sh-LINC00460) and control (sh-NC) T84 cells (Figure 3a) to explore the *in vivo* function of COL4A2-AS1 in CRC progression. HE and Ki67 were measured, and we observed that the tissues treated by shCOL4A2-AS1 cells showed karyotype fusion, shape change, and Ki67 reduction (Figure 3b). Moreover, COL4A2-AS1 knockdown resulted in decreased tumor volume and weight (Figure 3c,d). Consistent with cellular assay outcomes, LINC00460 knockdown also downregulated the LINC00460 expression in the T84 xenograft tumors (Figure 3e).

### COL4A2-AS1 knockdown suppressed aerobic glycolysis of the CRC cells

**Figure 4.**
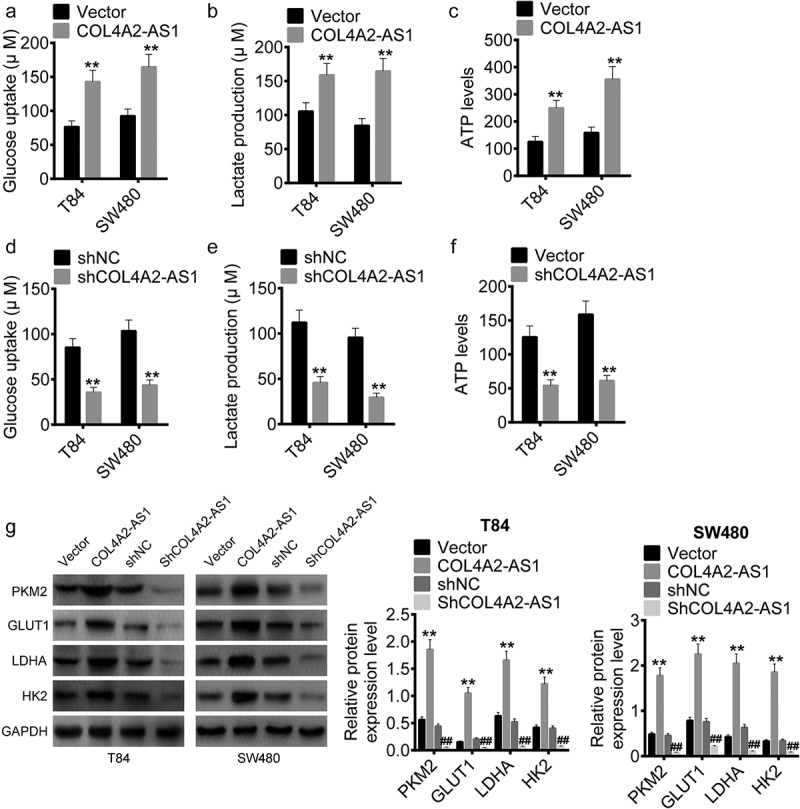
Down-regulation of COL4A2-AS1 expression inhibited aerobic glycolysis. (a–c): Overexpressed COL4A2-AS1 promoted lactate production, glucose uptake and intracellular ATP content of T84 and SW480 cells. (d–f): Downregulated expression of COL4A2-AS1 inhibited lactate production, glucose uptake and intracellular ATP content of T84 and SW480 cells. (g): Overexpressed COL4A2-AS1 decreased the protein expressions of PKM2, GLUT1, LDGA and HK2 of T84 and SW480 cells, while downregulation of COL4A2-AS1 produced opposite effects. **P < 0.01, ^##^*P* < 0.01

The role of COL4A2-AS1 in aerobic glycolysis were determined. The data revealed that in T84 and SW480 cells up-regulation of COL4A2-AS1 expression greatly increased glucose consumption, lactate production and ATP level (Figure 4a–c), which were noticeably inhibited by knocking down COL4A2-AS1 as predicted (Figure 4d–f). The mRNA expressions of glycolysis-related genes (PKM2, GLUT1, LDHA, and HK2) were also upregulated by overexpression of COL4A2-AS1 but downregulated by knocking down COL4A2-AS1 in T84 and SW480 cells when compared with those in the scramble (Figure 4g).

### COL4A2-AS1 bound to miR-20b-5p and downregulated miR-20b-5p expression

**Figure 5.**
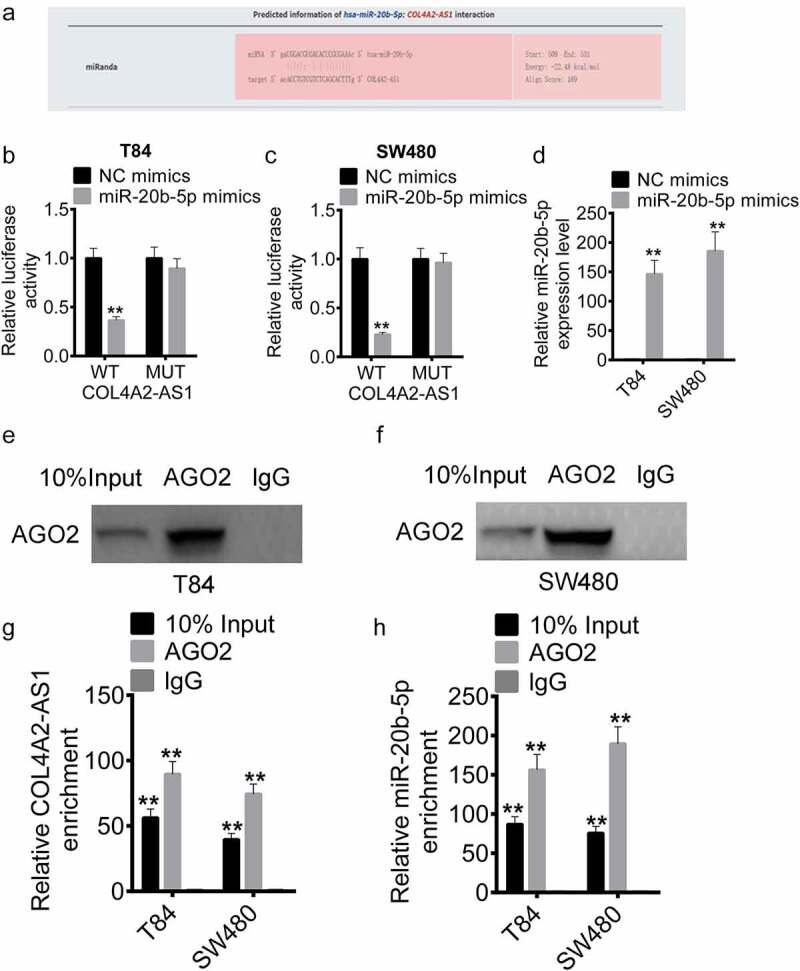
COL4A2-AS1 bound to miR-20b-5p and down-regulated miR-20b-5p expression. (a): LncACTdb predicted the binding region of miR-20b-5p and COL4A2-AS1. The effects of wt-COL4A2-AS1 or mut-COL4A2-AS1 on miR-20b-5p expression were detected by performing dual-luciferase reporter assay on T84 (b) and SW480 cells (c). (d): The expression level of miR-20b-5p was increased by miR-20b-5p mimic. (e–h): RIP experiments were performed on T84 and SW480 cells with Ago2 antibody, and the co-precipitated RNA was subjected to qPCR for COL4A2-AS1 and miR-20b-5p. **P < 0.01

The mechanism of action of COL4A2-AS1 was explored. LncACTdb prediction showed that COL4A2-AS1 had a mutual binding site with miR-20b-5p (Figure 5a). Subsequently, miR-20b-5p mimics were transfected into T84 and SW480 cells with COL4A2-AS1-wt or COL4A2-AS1-mut, respectively. The results demonstrated that miR-20b-5p overexpression significantly inhibited the luciferase activity of the COL4A2-AS1-wt plasmid but did not affect that of the COL4A2-AS1-mut plasmid in T84 and SW480 cells (Figure 5b,c). From RT-qPCR assay, it could be found that miR-20b-5p mimics successfully upregulated the expression of miR-20b-5p (Figure 5d). RNA immunoprecipitation (RIP) was conducted for AGO2 in T84 and SW480 cells, the expressions of endogenous COL4A2-AS1 and miR-20b-5p pulled-down from AGO2-expressed cells were determined by qRT-PCR analysis. Here, the results showed that COL4A2-AS1 and miR-20b-5p were expressed in the AGO2 pellet in comparison with those in the input control (Figure 5e–h).

### COL4A2-AS1 acted as a competing endogenous RNAs (ceRNA) of miR-20b-5p in the regulation of HIF1A expression

**Figure 6.**
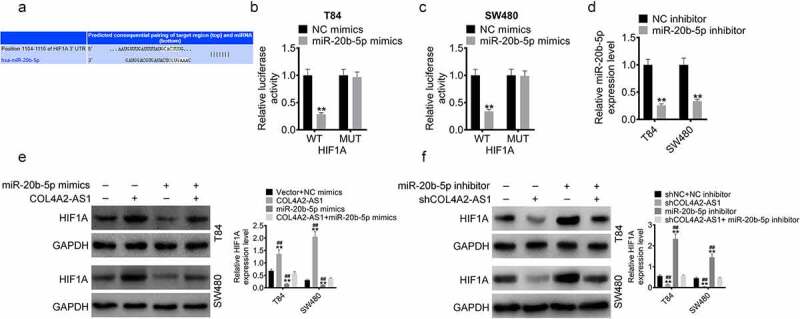
COL4A2-AS1 acted as a ceRNA of miR-20b-5p to regulate HIF1A expression. (a): TargetScan predicted the binding region between miR-20b-5p and the HIF1A 3ʹ-UTR. The effects of wt-HIF1A or mut-HIF1A on miR-20b-5p expression were detected by dual-luciferase reporter assay on T84 (c) and SW480 cells (d). (d): The expression of miR-20b-5p was decreased by miR-20b-5p inhibitor. (e): Western blot was performed for determining the expressions of HIF1A in T84 and SW480 cells transfected with control, COL4A2-AS1, miR-20b-5p mimics or COL4A2-AS1 + miR-20b-5p mimic. (f): Western blot was performed for determining the expression of HIF1A in T84 and SW480 cells transfected with control, shCOL4A2-AS1, miR-20b-5p inhibitor or shCOL4A2-AS1 + miR-20b-5p inhibitor. **P < 0.01, ^##^*P* < 0.01

To further examine underlying mechanism of action of COL4A2-AS1, TargetScan predicted that HIF1A was a potential target gene for miR-20b-5p (Figure 6a). Subsequently, miR-20b-5p mimics were transfected into T84 and SW480 cells with HIF1A-wt or HIF1A-mut. In T84 and SW480 cells, miR-20b-5p overexpression significantly inhibited the luciferase activity of the HIF1A-wt plasmid, but did not affect that of the HIF1A-mut plasmid (Figure 6b,c). Furthermore, we also determined the protein expressions of HIF1A of T84 and SW480 cells transfected with miR-20b-5p mimic, miR-20b-5p inhibitor or/and COL4A2-AS1 or/and shCOL4A2-AS1 as well as the transfection efficiency of miR-20b-5p inhibitor using RT-qPCR (Figure 6d). The data demonstrated that the protein expression of HIF1A was upregulated by COL4A2-AS1 but downregulated by miR-20b-5p mimic, moreover, the introduction of miR-20b-5p almost reversed the protein expression of HIF1A elevated by COL4A2-AS (Figure 6e). Similarly, the protein level of HIF1A was suppressed by shCOL4A2-AS1 but elevated by miR-20b-5p inhibitor, also, low-expressed miR-20b-5p almost reversed the protein level of HIF1A downregulated by shCOL4A2-AS (Figure 6f).

### HIF1A overexpression reversed the cell proliferation and glycolytic ability inhibited by knocking down COL4A2-AS1

**Figure 7.**
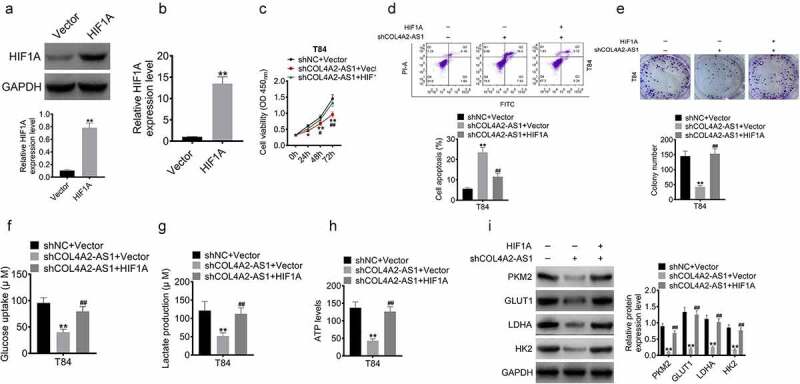
HIF1A overexpression reversed the inhibition of cell proliferation and glycolytic ability induced by knockdown of COL4A2-AS1. (a–b): The protein and mRNA levels of HIF1A in T84 cell transfected with HIF1A plasmid. (c): T84 cell viability was inhibited by inhibition of COL4A2-AS1 but was reversed by HIF1A. (d): Inhibition of COL4A2-AS1 promoted T84 cell viability, which was reversed by HIF1A. (e): Inhibition of COL4A2-AS1 inhibited T84 cell proliferation, which was reversed by HIF1A. (f–h): Inhibition of COL4A2-AS1 inhibited T84 cell lactate production, glucose uptake, and intracellular ATP content, which were reversed by HIF1A. (i): Inhibition of COL4A2-AS1 inhibited the protein expressions of PKM2, GLUT1, LDGA and HK2, which were reversed by HIF1A.*P < 0.05, **P < 0.01, ^#^*P* < 0.05, ^##^*P* < 0.01

To investigate the relationship between HIF1A and COL4A2-AS1, T84 cells were divided into three groups, namely, shNC+vector, shCOL4A2-AS1+ vector, and shCOL4A2-AS1+ HIF1A groups. Western blot and qRT-PCR performed to determine the miRNA and protein expressions of HIF1A showed that HIF1A expression was markedly upregulated in the cells transfected with HIF1A at protein and mRNA levels (Figure 7a,b). Subsequently, the transfected cells were harvested for MTT, apoptosis, and Transwell assays. Here, we found that the cell proliferation was reduced more sharply after downregulating COL4A2-AS1, which, however, was reversed by HIF1A (Figure 7c, d, and e). In addition, HIF1A significantly improved glucose consumption, lactate production, and ATP level after shCOL4A2-AS1 treatment (figure 7f, g, and h). The mRNA expressions of glycolysis-related genes (PKM2, GLUT1, LDHA, and HK2) were down-regulated by low-expressed COL4A2-AS1 but reversed by HIF1A (Figure 7i).

## Discussion

In this work, we found that lncRNA COL4A2-AS1 were upregulated in CRC tissues and cells. Silencing lncRNA COL4A2-AS1 suppressed CRC cell viability, proliferation, aerobic glycolysis, induced apoptosis, also inhibited tumor volume and weight. Furthermore, lncRNA COL4A2-AS1 demonstrated a tumor-promotion function by upregulating HIF1A through the downregulation of miR-20b-5p.

Currently, there are few studies conducted on the role of lncRNA COL4A2-AS1 in cancer. A previous study reported that lncRNAs are related to prognosis of breast cancer, which could therefore act as underlying prognosis biomarkers [12]. Based on this, the current study was the first to show that the level of lncRNA COL4A2-AS1 was clearly elevated in CRC tissues and cell lines. In 1924, Warburg discovered that even in the presence of oxygen, cancer cells consume large amounts of glucose at a high rate but supply ATP and produce lactate in a low efficiency. Such a phenomenon is known as the ‘Warburg effect’[31], which is also known as aerobic glycolysis, because glycolysis can occur even in the condition of sufficient oxygen. Aerobic glycolysis is controlled by many kinases including HK, PKM2, GLUT, and LDHA [32]. Robert A. Weinberg summarized that cancer cells widely feature energy reprogramming of the regulators of ‘aerobic glycolysis’[33]. Targeting tumor energy metabolism may also be a new therapeutic strategy. Interestingly, we found that knocking down COL4A2-AS1 inhibited the growth and aerobic glycolysis of CRC cells *in vivo* and *in vitro*. Those data indicated that COL4A2-AS1 overexpression contribute to CRC progression via regulating aerobic glycolysis.

It has been reported that lncRNAs could function as miRNA sponges, thereby regulating gene expressions at a post-transcriptional level. Based on the comprehensive results obtained from dual-luciferase reporter and RIP assays, COL4A2-AS1 was a sponge of miR-20b-5p in CRC cells to regulate HIF1A. Study confirmed that under-expressed miR-20b-5p plays a tumor-suppressive role in CRC [34–36]. Moreover, we also found that HIF1A expression was upregulated in a diverse range of human malignancies, including in CRC [37,38]. High-expressed HIF1A may be strongly correlated to the poor prognosis of hepatocellular carcinoma (HCC) patients [39]. High expression level of HIF1A indicates worse overall survival of CRC patients [40,41]. More importantly, HIF1 regulates the transcription of several key factors in glycolysis [42,43]. Thus, we speculated that COL4A2-AS1 was possibly related to miR-20b-5p and HIF1A. Online prediction tools showed that HIF1A directly targeted miR-20b-5p to promote miR-20b-5p expression, thus activating its transcription. These findings suggested pointed to the potential that COL4A2-AS1, miR-20b-5p and HIF1A formed a modulatory axis to affect the proliferation and glycolysis of CRC cells. According to the results of rescue assays, we confirmed that HIF1A was involved in the proliferation and aerobic glycolysis of CRC cells mediated by COL4A2-AS.

## Conclusion

This study did not examine whether COL4A2-AS1 inhibited aerobic glycolysis in xenogenic tumors. However, to conclude based on the obtained data, we are the first to reveal that the COL4A2-AS expression is upregulated in CRC tissues and cells. Through functional experiments, moreover, we also showed that COL4A2-AS acts as an oncogene in CRC to facilitate cell proliferation and aerobic glycolysis and affect tumor growth *in vivo*. Mechanically, COL4A2-AS may be sponged miR-20b-5p to regulate HIF1A expression in CRC. Those data demonstrated the clinical potential of COL4A2-AS to serve as a novel biomarker and potential therapeutic target for CRC.

## Highlights

1. LncRNAs COL4A2-AS1 expression was upregulated in CRC.
2. Silencing COL4A2-AS1 inhibited proliferation, aerobic glycolysis and promoted apoptosis of CRC.
3. MiR-20b-5p was sponged by COL4A2-AS1 and hypoxia-inducible factor 1 alpha subunit (HIF1A) in CRC cells.
